# Improving *Mycobacterium bovis* Bacillus Calmette-Guèrin as a Vaccine Delivery Vector for Viral Antigens by Incorporation of Glycolipid Activators of NKT Cells

**DOI:** 10.1371/journal.pone.0108383

**Published:** 2014-09-25

**Authors:** Manjunatha M. Venkataswamy, Tony W. Ng, Shalu S. Kharkwal, Leandro J. Carreño, Alison J. Johnson, Shajo Kunnath-Velayudhan, Zheng Liu, Robert Bittman, Peter J. Jervis, Liam R. Cox, Gurdyal S. Besra, Xiangshu Wen, Weiming Yuan, Moriya Tsuji, Xiangming Li, David D. Ho, John Chan, Sunhee Lee, Richard Frothingham, Barton F. Haynes, Michael W. Panas, Geoffrey O. Gillard, Jaimie D. Sixsmith, Birgit Korioth-Schmitz, Joern E. Schmitz, Michelle H. Larsen, William R. Jacobs, Steven A. Porcelli

**Affiliations:** 1 Department of Microbiology and Immunology, Albert Einstein College of Medicine, Bronx, New York, United States of America; 2 National Institute of Mental Health and Neuroscience, Bangalore, Karnataka, India; 3 Millennium Institute on Immunology and Immunotherapy, Facultad de Medicina, Universidad de Chile, Santiago, Chile; 4 Department of Chemistry and Biochemistry, Queens College of City University of New York, Flushing, New York, United States of America; 5 School of Biosciences, University of Birmingham, Edgbaston, Birmingham, United Kingdom; 6 School of Chemistry, University of Birmingham, Edgbaston, Birmingham, United Kingdom; 7 Department of Molecular Microbiology and Immunology, Keck School of Medicine, University of Southern California, Los Angeles, California, United States of America; 8 Aaron Diamond AIDS Research Center, Rockefeller University, New York, New York, United States of America; 9 Department of Medicine, Albert Einstein College of Medicine, Bronx, New York, United States of America; 10 Duke University Medical Center, Durham, North Carolina, United States of America; 11 Beth Israel Deaconess Medical Center, Harvard Medical School, Boston, Massachusetts, United States of America; 12 Howard Hughes Medical Institute, Albert Einstein College of Medicine, Bronx, New York, United States of America; Karolinska Institutet, Sweden

## Abstract

Recombinant *Mycobacterium bovis* bacillus Calmette-Guèrin (rBCG) has been explored as a vector for vaccines against HIV because of its ability to induce long lasting humoral and cell mediated immune responses. To maximize the potential for rBCG vaccines to induce effective immunity against HIV, various strategies are being employed to improve its ability to prime CD8^+^ T cells, which play an important role in the control of HIV infections. In this study we adopted a previously described approach of incorporating glycolipids that activate CD1d-restricted natural killer T (NKT) cells to enhance priming of CD8^+^ T cells by rBCG strains expressing an SIV Gag antigen (rBCG-SIV *gag*). We found that the incorporation of the synthetic NKT activating glycolipid α-galactosylceramide (α-GC) into rBCG-SIV *gag* significantly enhanced CD8^+^ T cell responses against an immunodominant Gag epitope, compared to responses primed by unmodified rBCG-SIV *gag*. The abilities of structural analogues of α-GC to enhance CD8^+^ T cell responses to rBCG were compared in both wild type and partially humanized mice that express human CD1d molecules in place of mouse CD1d. These studies identified an α-GC analogue known as 7DW8-5, which has previously been used successfully as an adjuvant in non-human primates, as a promising compound for enhancing immunogenicity of antigens delivered by rBCG.vectors. Our findings support the incorporation of synthetic glycolipid activators of NKT cells as a novel approach to enhance the immunogenicity of rBCG-vectored antigens for induction of CD8^+^ T cell responses. The glycolipid adjuvant 7DW8-5 may be a promising candidate for advancing to non-human primate and human clinical studies for the development of HIV vaccines based on rBCG vectors.

## Introduction

Recombinant mycobacteria have generated renewed interest in recent years as vectors for construction of vaccines against HIV/AIDS and other diseases. The currently used vaccine against tuberculosis (TB) in humans, *Mycobacterium bovis* bacillus Calmette-Guèrin (BCG), has been the preferred candidate for this purpose owing to the vast body of knowledge on its safety and immunogenicity, and the existence of standardized protocols for its testing and large-scale production [1]. Recombinant BCG (rBCG) strains expressing a wide variety of pathogenic bacterial and parasitic antigens have been investigated as candidate vaccines [2]. In fact, from the early days of HIV vaccine research, several antigens from simian and human immunodeficiency viruses (SIV and HIV) have been expressed in *M. bovis* BCG as an approach to the development of vaccine candidates [3]–[7]. This approach has been highly appealing for several reasons, including the known immunogenicity of BCG, its well established safety profile in humans, and the relative ease and low cost of its production and distribution. In addition, the use of rBCG expressing SIV or HIV antigens as a priming vaccine in heterologous prime-boost regimens has shown promising results in preclinical studies in murine and non-human primate models [3], [8]–[11].

Despite the encouraging preclinical results using rBCG as a priming vaccine, concerns remain that the existing rBCG strains are suboptimal with regard to their ability to generate sufficiently robust and long-lasting T cell responses. In particular, the priming of antigen specific CD8^+^ T cells against endogenous mycobacterial or transgene encoded antigens expressed by rBCG strains has generally been found to be of relatively low magnitude [9], [12], most likely reflecting the known immune evasion properties of pathogenic mycobacteria that enable them to suppress the development of effective T cell responses [13]. This has led in recent years to a variety of approaches to improve the immunogenicity of rBCG-based vaccines. For example, rBCG strains engineered to express either Listeriolysin-O from *Listeria monocytogenes* or perfringolysin from *Clostridium perfringens* have been generated to facilitate the translocation of mycobacterial antigens from phagosome to the cytosol, thereby improving access to the classical MHC class I antigen processing and presentation pathway in order to enhance CD8^+^ T cell priming [14], [15]. A pro-apoptotic rBCG strain with diminished superoxide dismutase activity has also been shown to prime T cell responses and protect against virulent *M. tuberculosis* challenge in mice more efficiently than a standard strain of BCG [16]. Thus, through genetic modifications of BCG vectors, there exist potential routes to the creation of highly effective new vaccines for TB, HIV and other infectious diseases.

Recently, there has been increasing interest in harnessing the immunomodulatory activity of natural killer T (NKT) cells to improve the T cell responses against vaccine antigens. Natural killer T cells are a conserved population of innate-like effectors that recognize specific glycolipid antigens presented by the MHC class I-like CD1d molecule. A major subset of these cells is strongly and specifically activated by glycolipids of the α-galactosylceramide (α-GC) family, which represent a potentially useful class of adjuvants that have shown promise in preclinical studies [17]. Stimulation of NKT cells by these glycolipids results in the production of a wide range of cytokines including IFNγ and IL-12p70, maturation of the CD8α^+^ dendritic cells (DCs) in the lymph nodes, and subsequent activation of NK and conventional T cells [18]. This cascade of immune reactions initiated by NKT cells in response to α-GC has been shown in mouse models to generate innate and adaptive immunity against a wide range of infections and tumors [17], [19], [20]. In addition, NKT cell activating glycolipids have been considered as potential vaccine adjuvants [21]–[25]. For example, α-GC and its synthetic analogue 7DW8-5 have been reported as effective adjuvants for DNA- or adenovirus-based vaccines against HIV [26], [27]. Similarly, a previous study from our laboratory showed that α-GC and its C-glycoside analogue α-C-galactosylceramide (α-C-GC) were able to enhance immunogenicity of *M. bovis* BCG against mycobacterial antigens and its protective efficacy against TB in mice [18]. In particular, we demonstrated that mice immunized with glycolipid modified BCG developed significantly increased antigen-specific CD8^+^ T cell priming responses.

In the present study, we examined the effects of incorporating NKT cell activating glycolipids on CD8^+^ T cell responses against epitopes of specific viral antigens expressed by recombinant BCG vectors. Using the adjuvant incorporation approach developed in our earlier study, we evaluated the effects of two potent synthetic α-GC analogues, α-C-GC and 7DW8-5, on CD8^+^ T cell priming in mice against a viral antigen expressed in recombinant BCG (rBCG). These studies monitored responses to an immunodominant epitope of an SIV Gag antigen as a viral immunogen expressed in rBCG, and also assessed the secondary response following boosting with a recombinant adenovirus vaccine expressing the homologous Gag protein. Our findings revealed substantial augmentation of CD8^+^ T cell responses and significant dose sparing effects for the glycolipid modified rBCG vaccines, allowing reduction in vaccine dose to levels more consistent with subcutaneous or intradermal delivery. In addition, studies carried out in human *CD1D* knock-in mice which more closely model the NKT cell response in humans, confirmed the enhanced CD8^+^ T cell priming effects seen in wild type mice. This partially humanized mouse model may provide a useful tool for guiding the selection of α-GC analogues to be used in future studies of rBCG vaccines in nonhuman primates or human subjects.

## Results

### Enhancement of CD8^+^ T cell responses by incorporation of α-GalCer into rBCG

**Figure 1:**
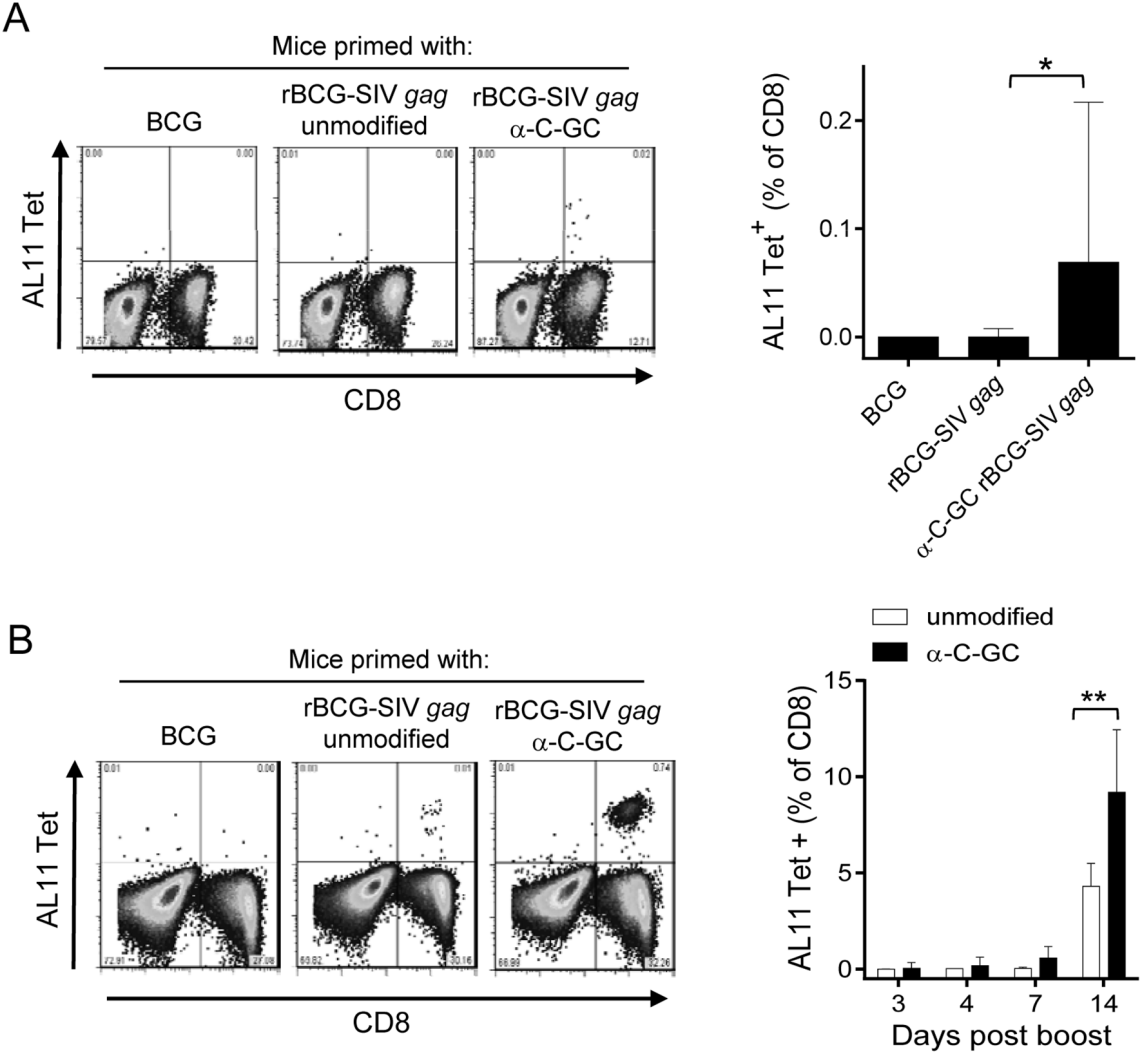
Enhanced primary and boosted responses to SIV Gag expressed in rBCG with incorporation of NKT cell activating glycolipids. C57BL/6 mice were primed i.v. with 10^7 ^CFU of *M. bovis* BCG, rBCG-SIV *gag*, or rBCG-SIV *gag* modified by incorporation of α-C-GC. CD8^+^ T cells specific for SIV Gag in peripheral blood samples were enumerated by flow cytometry of lymphocytes stained with H2D^b^/AL11 tetramers specific for an immunodominant Gag epitope. (A) Primary responses were analyzed at 14 days after priming. Plots on left show representative profiles of tetramer staining of CD8^+^ T cells (for complete gating strategy, see supplementary Fig. S2). Graph on right shows medians and interquartile ranges for groups of similarly immunized mice (n = 5 mice per group). *P<0.05 (Kruskal-Wallis followed by Mann-Whitney test with Bonferroni correction). (B) Secondary responses to SIV Gag in mice boosted with rAd5-SIV *gag* (10^7^ VP i.m.) eight weeks after priming. Plots on left show representative tetramer staining on day 7 after boosting. Graph on right shows medians and interquartile ranges at the indicated day after boosting for groups of mice (n = 5 per group) primed with rBCG-SIV *gag* without glycolipid modification (unmodified, open bars), or with rBCG-SIV *gag* with incorporation of α-C-GC (filled bars). **P<0.01 (Mann-Whitney test).

In previous studies focused on the generation of responses to endogenous mycobacterial antigens, we showed that specific CD8^+^ T cell responses were significantly enhanced by incorporation of NKT cell activating glycolipids into a standard BCG vaccine strain [28]. To extend these findings to rBCG vectors expressing viral antigens, we used a BCG strain engineered to express an SIV-Gag antigen (rBCG-SIV *gag*). Initial studies were performed by priming C57BL/6 mice intravenously (i.v.) with a relatively high dose (10^7 ^CFU) of rBCG-SIV *gag* that was either unmodified or modified by direct incorporation of the C-glycoside α-GC analogue (α-C-GC). Subsequently, animals were bled at several time points and peripheral blood lymphocytes (PBLs) were stained with the H2D^b^/AL11 tetramer which identifies CD8^+^ T cells specific for the immunodominant SIV Gag epitope. In several experiments, we observed that the primary CD8^+^ T cell responses in mice receiving unmodified rBCG-SIV *gag* vaccine were undetectable, whereas these responses were consistently detected when the glycolipid modified vaccine was administered (Fig. 1A).

Although the glycolipid adjuvant clearly facilitated primary antigen specific CD8^+^ T cell responses, these were of relatively low magnitude. Therefore, we examined the effect of boosting these responses by administering a suboptimal dose (10^7^ viral particles (VP)) of replication incompetent recombinant adenovirus (serotype 5) expressing the full length SIV Gag protein (rAd5-SIV *gag*) by the intramuscular (i.m.) route. Levels of circulating CD8^+^ T cells specific for SIV Gag were monitored by staining of PBLs with H2D^b^/AL11 tetramers. Starting at day 3 after boosting, a detectable difference in AL11 tetramer positive T cell population was observed between mice that were primed with unmodified and α-C-GC modified rBCG-SIV *gag*, reaching a significant difference after day 14 post boosting (Fig. 1B). Only by day 14 did AL11 specific CD8^+^ T cell responses become detectable in the mice primed with unmodified rBCG-SIV gag, although these responses were still higher in mice primed with the glycolipid modified rBCG-SIV *gag*. These results provided evidence that the administration of α-C-GC adjuvant in direct physical association with the priming vaccine had a favorable impact on the induction of antigen specific CD8^+^ T cell responses, resulting in a pool of primed CD8^+^ T cells that were able to respond rapidly to the booster vaccine.

### Effects of glycolipid modification on subcutaneous priming with rBCG

**Figure 2:**
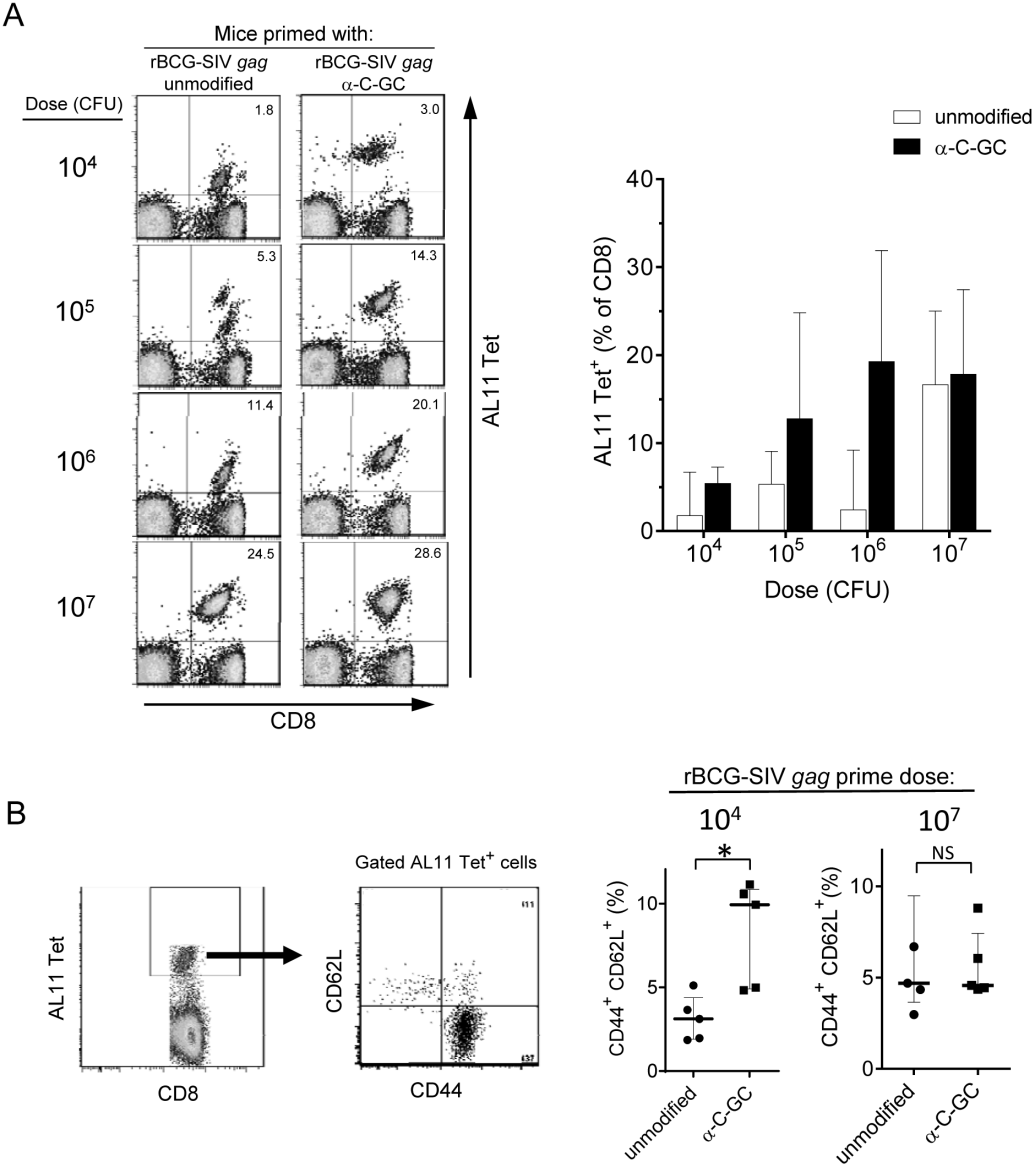
Dose-sparing effect of α-C-GC on CD8^+^ T cell priming and increased expression of central memory phenotype. (A) C57BL/6 mice were immunized s.c. with varying doses of rBCG-SIV *gag* (10^4^, 10^5^, 10^6^, and 10^7 ^CFU) with incorporation of α-C-GC or without glycolipid incorporation (unmodified). All animals were boosted 8 weeks later with 10^7^ VP i.m. of rAd5-SIV *gag*, and PBL were analyzed for AL11 tetramer staining at day 14 post-boost. Plots on left show Gag-specific CD8^+^ T cell responses in representative mice. The percentages of tetramer positive cells are indicated in the upper right quadrants. The graph on the right shows medians and interquartile ranges for groups of 4 mice each. Open bars indicate mice that were primed with unmodified rBCG-SIV *gag*, and filled bars indicate mice primed with α-C-GC modified rBCG-SIV *gag*. *P* = 0.0571 between unmodified and α-C-GC rBCG-SIV *gag* for both 10^5^ and 10^6 ^CFU; Kruskall-Wallis followed by Mann-Whitney test with Bonferroni correction). (B) Long term Gag-specific CD8^+^ memory T cells were analyzed for expression of CD62L and CD44 as shown on the left. The graphs on the right show levels of central memory AL11-specific CD8^+^ T cells in PBL of mice (medians and interquartile ranges for groups of 5 mice) primed s.c. with two different doses (10^4^ or 10^7 ^CFU) of rBCG-SIV gag that were either unmodified or modified by incorporation of α-C-GC. All animals were boosted with 10^7^ VP i.m. of rAd5-SIV *gag* eight weeks after priming, and spleens were harvested six months later for analysis. *P<0.05 (Mann-Whitney test).

We next examined whether the effects of the glycolipid modification observed with i.v. immunization could also be seen after subcutaneous (s.c.) priming with rBCG-SIV *gag*. In initial experiments, a relatively high priming dose (5×10^6 ^CFU) of rBCG-SIV *gag* either with or without incorporation of α-C-GC was administered s.c., followed by boosting i.m. with a suboptimal dose (10^7^ VP) of rAd5-SIV *gag*. This revealed a clear priming of AL11 specific CD8^+^ T cell responses with s.c. injection of the glycolipid modified rBCG, but not with the unmodified rBCG vaccine (data not shown). This prompted us to examine a range of doses of rBCG for s.c. priming to determine the extent of the dose-sparing effect that could be achieved by glycolipid modification. Subcutaneous priming was done using 10^4^, 10^5^, 10^6^ or 10^7 ^CFU of α-C-GC modified or unmodified rBCG-SIV *gag*, followed by boosting with rAd5-SIV *gag* eight weeks later. This showed that glycolipid adjuvant incorporation into rBCG promoted a consistent trend toward higher secondary CD8^+^ T cell responses 14 days after boosting with rAd5-SIV *gag* even at a priming dose as low as 10^5 ^CFU, while the unmodified rBCG required 10^7 ^CFU to give comparable priming (Fig. 2A, bar graph). This suggested a 10 to 100 fold dose sparing effect on SIV Gag specific CD8^+^ T cell priming attributable to glycolipid modification of the rBCG-SIV *gag* vaccine.

We also assessed the impact of rBCG dose and glycolipid incorporation on the development of long term memory CD8^+^ T cell responses. Mice were primed with 10^4^, 10^5^, 10^6^ or 10^7 ^CFU of α-C-GC modified or unmodified rBCG-SIV *gag*, and then boosted 8 weeks later with rAd5-SIV *gag*. Six months after boosting, spleens were harvested and expression of CD44 and CD62L was analyzed on CD8^+^ cells stained with H2D^b^/AL11 tetramers (Fig. 2B). No significant differences were observed between the groups in the fraction of Gag-specific CD8^+^ T cells displaying an effector memory phenotype (CD44^+^ CD62L^neg^; data not shown). However, we observed a trend toward an increase in the fraction of central memory cells (CD44^+^ CD62L^+^) in animals primed with the glycolipid modified rBCG. This rose to a level that was statistically significant only in animals immunized with the lowest dose (10^4 ^CFU) of the glycolipid modified rBCG (Fig. 2B). This result suggested that the formation of stable long term memory CD8^+^ T cells was promoted by relatively low doses of the priming immunogen when combined with incorporation of α-C-GC as an adjuvant during priming.

### Effects of glycolipid modified rBCG on NKT cell anergy

**Figure 3:**
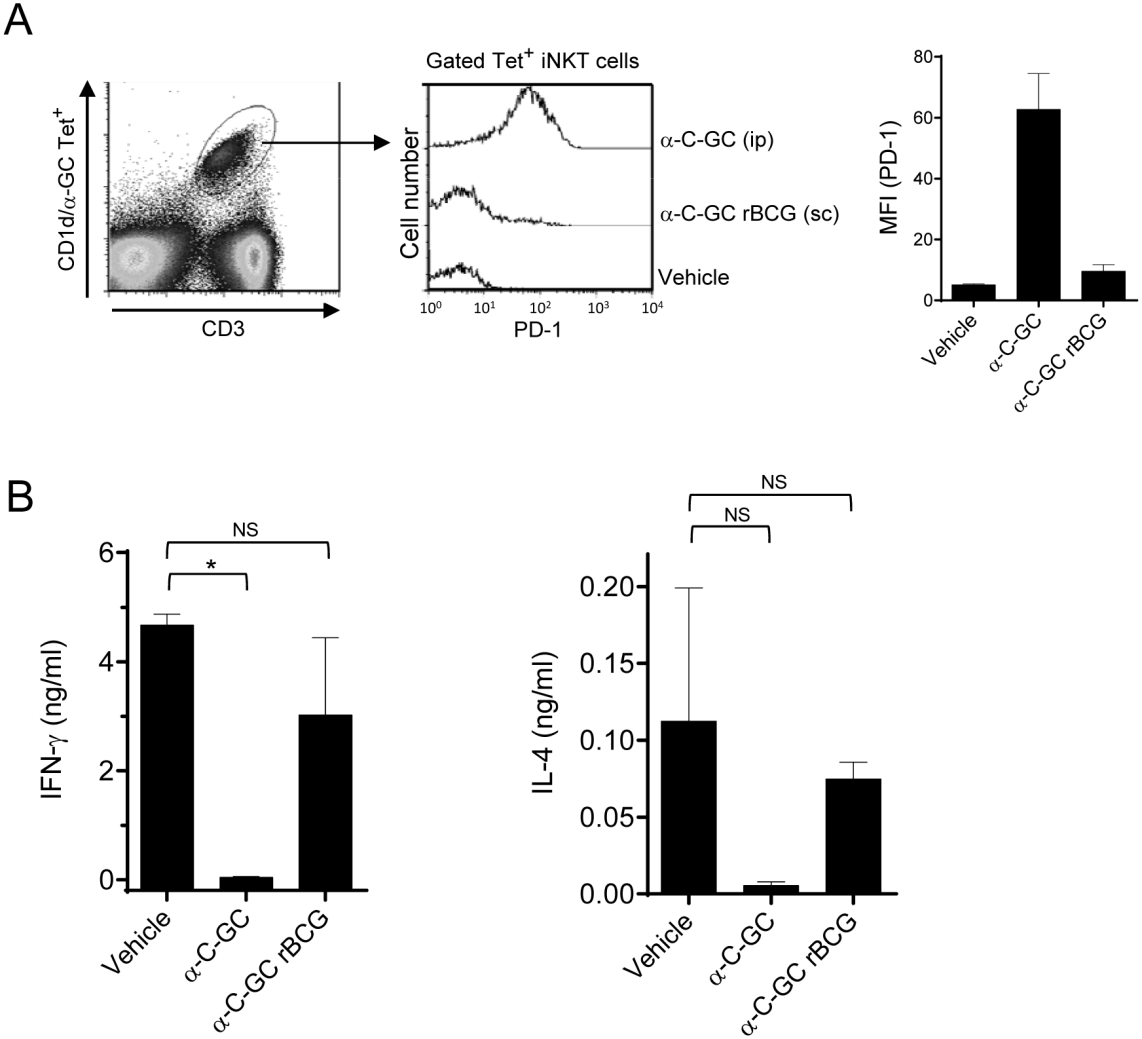
Absence of NKT cell anergy following immunization with glycolipid modified rBCG. Mice were injected i.p. with 4 µg of free glycolipid α-C-GC, or s.c. with 5×10^6 ^CFU of rBCG-SIV *gag* modified with α-C-GC. After 7 days, splenocytes were analyzed for expression of PD-1 on α-GC-loaded CD1d tetramer^+^ NKT cells as shown in (A), or were re-stimulated *in vitro* for 48 hours with α-GC for measurement of recall cytokine responses by capture ELISA of IFN-γ and IL-4 in the culture supernatant (B). Graphs show median values with interquartile ranges for groups of three mice each. *P<0.05; NS, not significant (Kruskal-Wallis followed by Mann-Whitney test with Bonferroni correction).

NKT cells are known to undergo anergy following a single injection of α-GC in mice and to remain in the non-responsive state for more than a month after being stimulated in this way *in vivo*. It is thus of interest to determine whether the α-C-GC modified BCG vaccine vector induces NKT cell anergy when given by a standard vaccination route (i.e., subcutaneously), which might have undesirable effects on post-vaccination immune status. Induction of anergy in NKT cells following systemic stimulation with α-GC has been correlated with increased surface expression of PD-1 [29]. Therefore, we analyzed PD-1 expression on the surface of CD1d/α-GC tetramer positive NKT cells in the spleens of mice following s.c. administration of 5×10^6 ^CFU of α-C-GC modified rBCG-SIV *gag*. This revealed no significant induction of PD-1, in contrast to the NKT cells in spleens of mice receiving injections of free α-C-GC in aqueous vehicle which showed increased expression of PD-1 (Fig. 3A). We also studied *ex vivo* recall responses of NKT cells to α-GC in the spleen seven days after administering either free glycolipids (α-GC or α-C-GC in aqueous vehicle) or rBCG-SIV *gag* modified with these glycolipids. Splenocytes from these mice were re-stimulated in culture with α-GC for 48 hours, and levels of IFN-γ and IL-4 were measured in the supernatant. Cytokine secretion in response to α-GC was intact in mice that had received the glycolipid modified rBCG immunization. In contrast, these responses were markedly diminished in the mice previously injected with the free glycolipids, consistent with the induction of NKT cell anergy (Fig. 3B). These results indicated that s.c. administration of glycolipid modified rBCG did not induce anergy of NKT cells.

### Modification of rBCG-SIV *gag* using an α-GC analogue optimized for human NKT cells

The effects of α-C-GC observed in our experiments were consistent with the previously demonstrated high potency of this glycolipid as an activator of NKT cells in mice. However, it has been shown that α-C-GC is less active for stimulation of human NKT cells, and may not be an optimal choice for use as an adjuvant in primates [30]. Recently, an α-GC analogue known as 7DW8-5 has been reported to be a potent activator of human NKT cells that performs well as an experimental vaccine adjuvant in nonhuman primates [31]. Similar to α-C-GC, the *in vivo* cytokine response to 7DW8-5 following systemic administration in mice reveals a bias toward strong IFN-γ production with relatively low, transient IL-4 secretion (Fig. S3) [32]. These properties have stimulated substantial interest in the development of 7DW8-5 as an adjuvant for human vaccination [33]. We undertook studies to evaluate 7DW8-5 as a candidate adjuvant for rBCG vaccines, and assessed its activities in wild type mice and in a partially humanized human CD1d knock-in mouse model.

**Figure 4:**
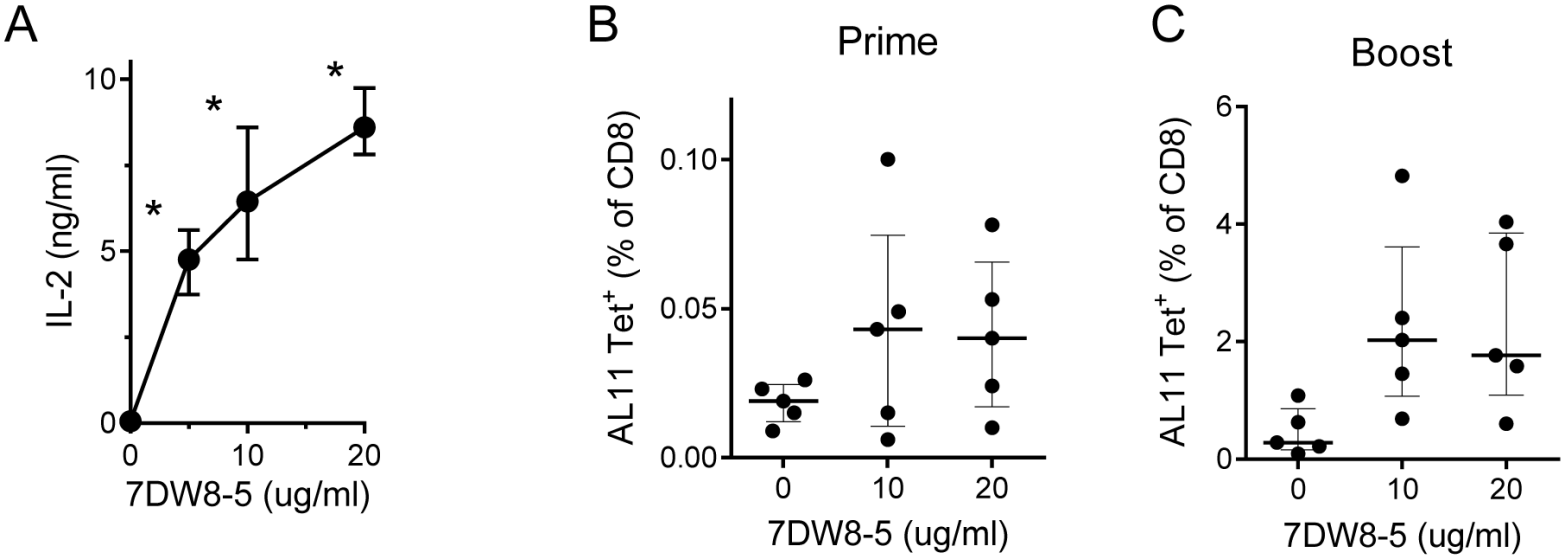
Enhanced Gag-specific response with incorporation of α-GC analogue 7DW8-5 into rBCG. (A) To determine the optimal concentration of glycolipid 7DW8-5 for incorporation into rBCG-SIV *gag*, a range of concentrations up to 20 µg/ml were tested. Mouse bone marrow dendritic cells were infected with mock treated or glycolipid modified rBCG-SIV *gag* at a multiplicity of infection of 10∶1, and cultured with NKT cell hybridoma DN32.D3. Levels of IL-2 in the culture supernatants were assayed 24 hours later by ELISA to assess glycolipid incorporation. Median values with ranges are shown for triplicate samples. C57BL/6 mice were primed with 10^7 ^CFU i.v. of unmodified rBCG-SIV *gag* or rBCG-SIV *gag* incorporated with 10 or 20 µg/ml of 7DW8-5, and analyzed for Gag-specific CD8^+^ T cells by AL11 tetramer staining of PBL samples obtained 10 days after priming (B) or 10 days after boosting (C) with 10^7^ VP i.m. of rAd5-SIV *gag*. Values for individual mice are shown as the percentage of AL11 tetramer stained cells in the total CD8^+^ T cell fraction, and median values are indicated with interquartile ranges. *P<0.05 (Kruskal-Wallis followed by Mann-Whitney test with Bonferroni correction).

In initial studies with 7DW8-5, we analyzed incorporation into live rBCG-SIV *gag* cultures using concentrations of the glycolipid ranging from 5 to 20 µg/ml, and tested the ability of the modified bacteria to stimulate an NKT cell hybridoma (Fig. 4A). We found a dose-dependent enhancement of hybridoma responses consistent with stable incorporation of 7DW8-5 into the live mycobacteria, with concentrations of 10 and 20 µg/ml delivering optimal responses. To assess the effect on priming of CD8^+^ T cell responses to the immunodominant Gag epitope, 10^7 ^CFU of rBCG-SIV *gag* modified with various concentrations of 7DW8-5 were injected i.v. into C57BL/6 mice. Levels of CD8^+^ cells staining with the H2D^b^/AL11 tetramer were measured in PBL samples ten days later. We observed enhanced priming of Gag-specific CD8^+^ T cells in mice receiving rBCG-SIV *gag* modified with the higher concentrations of 7DW8-5 (Fig. 4B). To determine if the enhanced CD8^+^ T cell priming could generate improved responses after boosting, these mice were injected with rAd5-SIV *gag* (10^7^ VP i.m.) six weeks after initial priming. This revealed increased Gag-specific CD8^+^ T cell responses at 10 days after boosting in animals primed with the 7DW8-5 modified rBCG-SIV *gag* (Fig. 4C). In these studies, 10 µg/ml was found to be the most efficient concentration of 7DW8-5 for incorporation of the glycolipid into rBCG-SIV *gag*, resulting in the highest mean CD8^+^ T cell responses following both priming (Fig. 4B) and boosting (Fig. 4C).

### Activity of the glycolipid adjuvant 7DW8-5 in a humanized mouse model

**Figure 5:**
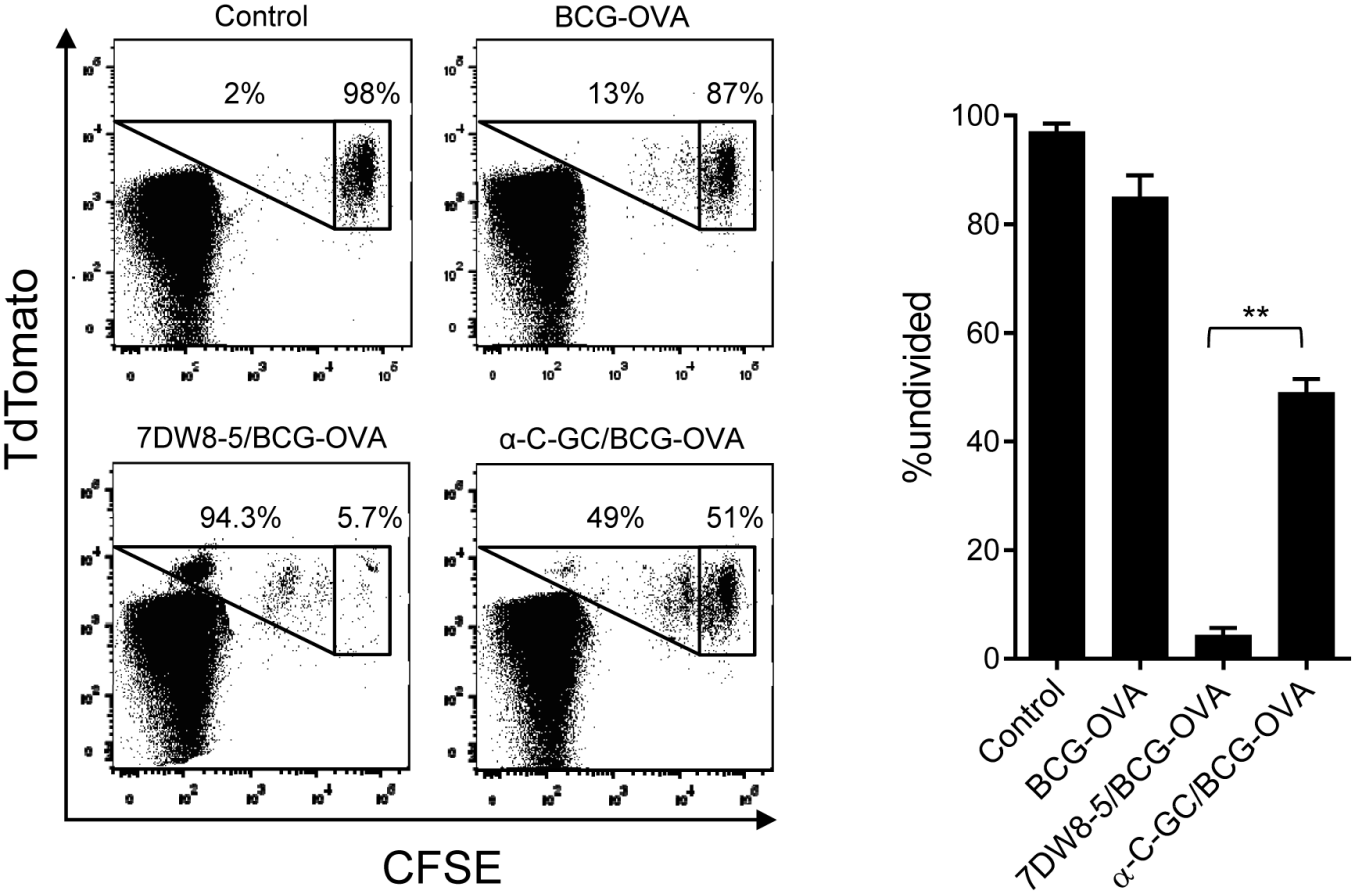
Comparison of 7DW8-5 with α-C-GC in human CD1d knock-in mice. Purified CD8^+^ T cells from tdTomato-Tg/OT-I TCR-Tg mice labeled with CFSE were transferred into recipient humanized CD1d knock-in mice that were subsequently injected s.c. with PBS (Control), rBCG-OVA, or rBCG-OVA modified with either 7DW8-5 or α-C-GC. After 6 days, splenocytes were harvested and CFSE fluorescence of the transferred population was determined by gating on the B220^Neg^ CD8^+^ tdTomato^+^ lymphocytes. Representative plots showing CFSE staining and dilution are shown; OVA-specific CD8^+^ T cells are enclosed in the outlined areas of the dot plots, with residual undivided cells (i.e., without CFSE dilution) in the right hand boxes. The graph summarizes the results as the medians and interquartile ranges of the percentages of remaining undivided OVA-specific CD8^+^ T cells for groups of mice (n = 5 per group). **P<0.001(Kruskal-Wallis followed by Mann-Whitney test with Bonferroni correction). These results are representative of two separate experiments.

A human CD1d knock-in (hCD1d-KI) mouse line has recently been described, providing a useful partially humanized mouse model for the assessment of NKT cell based therapeutics and adjuvants [34]. In these mice, the coding sequence of human CD1d has been inserted in place of the mouse gene, leading to expression of human CD1d in a normal pattern of tissue distribution and at levels similar to murine CD1d in wild type mice. Compared to C57BL/6 and other standard mouse strains, these animals develop a functional repertoire of NKT cells with frequency and distribution much more similar to normal humans. To determine if 7DW8-5 could serve as an effective adjuvant when incorporated into rBCG in this humanized mouse model, we studied CD8^+^ T cell priming using an adoptive transfer system. Naïve CD8^+^ T cells were isolated from mice expressing the red fluorescent protein tandem dimer tomato (TdT) and TCR transgenes specific for the ovalbumin (OVA) peptide SIINFEKL. These were labeled with carboxyfluorescein diacetate succinimidyl ester (CFSE), and adoptively transferred into hCD1d-KI mice. These animals then received s.c. immunization with BCG expressing ovalbumin (rBCG-OVA) that was either unmodified or modified with 7DW8-5 or α-C-GC. After 6 days, splenocytes were harvested and analyzed for proliferation of OVA-specific CD8^+^ T cells by flow cytometry (Fig. 5). An increase in CD8^+^ T cell proliferation was observed with 7DW8-5 compared to α-C-GC modified rBCG-OVA, consistent with higher activity of 7DW8-5 in this partially humanized mouse model.

## Discussion

We previously demonstrated that CD8^+^ T cell responses to endogenous antigens of *M. bovis* BCG can be significantly enhanced by incorporation of NKT cell activating glycolipids such as α-GC and α-C-GC [28]. In the current study, we have applied this approach to improving CD8^+^ T cell responses to a retroviral antigen expressed in recombinant BCG. Using an SIV Gag protein as the model antigen and the standard Danish strain of BCG as the live-attenuated delivery vector, we demonstrated significant improvements in immunogenicity directed to the specific MHC class I presented immunodominant epitope of SIV Gag by incorporation of the NKT cell activating glycolipids α-C-GC and 7DW8-5. This method allows for simultaneous delivery of protein antigens and NKT cell activating ligands by direct infection of antigen presenting cells (APCs). These include CD8α^+^ DCs, which in mice are known to be the major APC involved in both NKT cell responses and cross-priming of conventional CD8^+^ T cells [35]. As previously shown, mycobacteria including BCG do not generally express strong activators of NKT cells [36]. Our approach of directly incorporating synthetic CD1d-presented glycolipids provides a relatively simple approach to correcting this deficiency, thus improving the overall potential of BCG as a vaccine delivery vector. The ability of this approach to enhance responses of CD8^+^ T cells against specific antigens of viruses or other pathogens expressed in rBCG strains could potentially assist a wide range of vaccine targets. For example, relevant to the particular case of the Gag antigen used in the current study, it is well documented that Gag specific CD8^+^ T cell responses are strongly associated with natural control of HIV infection in humans and with control of viral set point in experimental SIV infection in macaques [37].

Although the levels of circulating CD8^+^ T cells specific for the immunodominant SIV Gag AL11 epitope using glycolipid modified rBCG-SIV *gag* were relatively low after priming, these responses showed robust and rapid expansion following boosting with a sub-maximal dose of nonreplicating adenovirus expressing the homologous SIV Gag antigen. The accelerated secondary response observed in mice primed with adjuvanted vaccines in this prime-boost model suggests that the CD8^+^ T cells generated by this regimen may have properties that reduce the delay in the response of HIV-specific central memory CD8^+^ T cells upon exposure to the virus. This acceleration of the response could help to overcome the kinetic mismatch between viral replication and the generation of effective immune control, which may be partly responsible for the failure to clear infection by viruses that attack the immune system directly such as SIV or HIV [38]. Furthermore, with incorporation of the potent Th1-biasing α-C-GC glycolipid, the immune enhancing role of the adjuvant on the secondary response was retained when the priming vaccine was delivered subcutaneously. Using this route of vaccination, the incorporation of the glycolipid adjuvant gave a 10- to 100-fold dose-sparing effect. This may assist the development of vaccination regimens that minimize local or systemic adverse effects without compromising immunogenicity. It should be noted that our analyses have so far focused only on the effects of glycolipid modification of rBCG on CD8^+^ T cell responses in blood and spleen. Further studies will be needed to determine how this approach influences T cell responses in other relevant tissues such as lymph nodes and various mucosal sites.

It is noteworthy that early phase clinical studies have shown that i.v. injection of α-GC causes rapid immune activation in humans without significant toxicity [39], [40]. However, one potential problem with α-GC that has been frequently observed in mouse models is its property of inducing long-term anergy of NKT cells, lasting for a month or more after systemic administration of α-GC [41], [42]. This results in the NKT cells being unresponsive to a second stimulation with α-GC either *in vivo* or *in vitro*, creating a prolonged host immune defect. However, when administered in physical association with rBCG, we observed no evidence of systemic anergy or unresponsiveness of NKT cells. Thus, splenic NKT cells from mice injected with α-C-GC modified rBCG-SIV *gag* seven days earlier were reactive to α-GC re-stimulation *in vitro*. We have not yet established how this approach avoids the problem of NKT cell anergy, although one possibility is that association with the bacilli restricts delivery of the glycolipid to antigen presenting cells that are incapable of inducing anergy. Whatever the mechanism, this feature of the response to glycolipid modified rBCG raises the possibility of regimens involving the repeated use of NKT cell activators for both priming and boosting of responses.

Selection of the optimal chemical form of α-GC for various different applications has been an important theme of recent work in the field of NKT cell-based therapeutics [43]. Although modification of rBCG with α-C-GC was potent in augmenting Gag-specific CD8^+^ T cell responses in wild type mice, this synthetic glycolipid has been found to be less active for stimulation of human NKT cells and may not be optimal for clinical translation into humans [30], [44]. Recent work has identified other NKT cell activators, in particular the α-GC analogue 7DW8-5, as more promising agents for development as human vaccine adjuvants or immunomodulators [27], [31], [33]. Although we have not developed methods for directly quantifying the amount of α-C-GC or 7DW8-5 that are incorporated into rBCG, based on previous work we anticipate that approximately 20–30% of the glycolipid added to growing cultures becomes stably associated with the bacilli [28]. In comparisons of α-C-GC and 7DW8-5 directly for modification of rBCG, we observed that the former was more potent for eliciting Gag-specific CD8^+^ T cell responses in wild type mice. However, in hCD1d-KI mice, in which presentation occurs through human CD1d only, 7DW8-5 showed superior adjuvant effects in our rBCG priming model. This is consistent with published findings on 7DW8-5, which showed it to be among the most active α-GC analogues tested in non-human primates and in cell culture with human cell lines [27]. Both α-C-GC and 7DW8-5 have been described to stimulate a Th1-biased immune response in mice [27], [45], [46], which is a quality that is generally associated with improved CD8^+^ T cell cross priming [43].

In summary, we have shown in this study that incorporation of NKT cell activating glycolipids provides adjuvant effects that facilitate stronger CD8^+^ T cell responses against a viral antigen expressed in rBCG. Although SIV Gag has been used as a convenient model antigen for these studies, this antigen and its homologue in HIV are known to be important targets for virus specific CD8^+^ T cell responses that suppress circulating viral levels and slow disease progression [37]. Thus, direct clinical applications can be envisioned for glycolipid modified rBCG strains as vaccines for simultaneously stimulating beneficial immunity against both tuberculosis and HIV. This approach should be applicable for enhancing antigen specific CD8^+^ T cell responses to live attenuated rBCG vaccine vectors expressing a wide variety of antigens, including other antigens of HIV or expressed by other relevant pathogens. We conclude that the NKT cell based adjuvant, in addition to enhancing immunogenicity of the rBCG vector, provides significant practical advantages as it enables significant reduction in the priming vaccine dose, and improves responses following vaccination by a clinically feasible route. Since rBCG strains can be constructed and produced with relative ease and low cost, this approach to improving their immunogenicity has potential applications for novel vaccines against a range of infectious diseases.

## Materials and Methods

### Antibodies and staining reagents

Antibodies for murine IL-2, IFNγ and IL-4 capture ELISA were purchased from BD Biosciences. Antibodies used for flow cytometry (all from BD Biosciences) included anti-mouse CD45R/B220-PerCP, anti-mouse CD45R/B220-FITC, anti-mouse CD3-FITC, anti-mouse CD3-V500, anti-mouse CD8α-APC, anti-mouse CD8α-Pacific Blue, anti-mouse CD44-FITC,anti-mouse CD62L-APC, and anti-mouse PD1-PE. BluVid viability dye (Invitrogen) was used for live/dead staining. The APC-conjugated α-GC loaded mCD1d tetramer and PE-conjugated AL11/H2D^b^ tetramer specific for the SIV Gag immunodominant epitope (AAVKNWMTQTL) were obtained from the NIH Tetramer Core Facility.

### Preparation of cell suspensions and flow cytometry

To obtain peripheral blood lymphocytes (PBL), approximately 300 µl of blood was obtained by retro-orbital plexus puncture and collected into 2 ml of RPMI-1640 containing 10 U/ml of heparin (Sigma). Samples were centrifuged at 650×g for 10 minutes at 4°C, supernatants were aspirated and 2 ml of red blood cell lysing buffer (Sigma) was added. After 2 minutes at room temperature, 10 ml of PBS was added, and cells were collected by centrifugation and resuspended in FACS staining buffer (PBS+2% FCS and 0.05% NaN_3_) for staining with fluorochrome labeled antibodies and tetramer reagents. Spleens were collected aseptically, homogenized in 10 ml of RPMI-1640, and treated with red blood cell lysing buffer similar as for blood samples except that 40 ml of PBS was used for washing and removal of the red blood cell lysing buffer. PBL were dispensed into wells of 96-well round bottom plates (Corning), washed once with PBS, and live/dead staining was performed with the Blue Live/Dead kit (Invitrogen) in PBS on ice for 20 minutes according to the manufacturer’s instructions. The cells were pelleted at 650×g for 2 minutes at 4°C and washed with PBS. Samples were then incubated with the specific fluorochrome labeled antibodies or tetramers of interest, along with the FcγRII/III blocking antibody 2.4G2 in FACS buffer (PBS, 2% FBS, 0.05% sodium azide) for 20 minutes at room temperature. Flow cytometry analysis was carried out using FACSCalibur and LSR II flow cytometers (BD Biosciences), and data were analyzed using FlowJo software (Treestar).

### Recombinant BCG and adenovirus strains

The recombinant BCG strain expressing ovalbumin (BCG-OVA) has been described previously [28]. *Mycobacterium bovis* BCG Danish 1331 (Statens Serum Institute) was used for construction of rBCG expressing SIV Gag, which will be described in detail elsewhere (S. Lee, et al., manuscript in preparation). Briefly, the full-length coding sequence of the *gag* gene of SIV strain Mac239 was amplified by standard RT-PCR. This was ligated into the non-integrating mycobacterial expression plasmid pSL7 (S.L. and W.R.J., unpublished), in which the *gag* sequence was fused to the *M. tuberculosis hsp60* gene promoter and upstream translation initiation site of the *M. tuberculosis* 19 kDa lipoprotein gene (*LpqH,* Rv3763). Transformants of BCG were selected using 50 µg/ml apramycin, cloned by selection of isolated colonies and screened for expression of the full length SIV Gag protein by Western blotting. Recombinant BCG strains were grown in Middlebrook 7H9 media (Becton Dickinson) supplemented with 0.05% tyloxapol (Sigma) and 10% oleic albumin dextrose catalase enrichment (OADC) (Becton Dickinson). Media were supplemented with 50 µg/ml apramycin for rBCG-SIV *gag*, or with 50 µg/ml kanamycin (Sigma) for BCG-OVA. The recombinant adenovirus expressing Mac239 SIV *gag* (rAd5-SIV*gag,* generously provided by Dr. Jason Gall, GenVec, Gaithersburg, MD) was constructed in a serotype 5 virus with deletions in the E1 and E3 regions to render it replication incompetent [47]. Methods used for production of adenoviral stocks and determination of viral particle (VP) titers have been previously described [47], [48].

### Incorporation of glycolipids into rBCG

The glycolipids α-GC, α-C-GC and 7DW8-5 were synthesized as previously described [27], . The chemical structures of these glycolipids are shown in Fig. S1. Incorporation of the glycolipids into live BCG was done using a previously published approach [28] with minor modifications. The bacteria were grown to mid-log phase (OD_600_ of 0.6), harvested and washed with PBS (Life Technologies) containing 0.05% tyloxapol (Sigma). The bacterial pellet was resuspended and diluted in 7H9 medium without OADC to give an OD_600_ of 3.0 (∼9×10^8 ^CFU/ml). Glycolipids were suspended at 10X the desired final concentrations in 7H9 medium containing 20% DMSO and 0.5% tyloxapol. To suspend the glycolipids in this aqueous medium, solutions were sonicated for 5 minutes in a bath sonicator (Branson), followed by mixing for 1 minute in a vortex mixer and heating to 80°C for 2 minutes. The bacterial suspension was combined with an equal volume of glycolipid suspension, and this was then diluted tenfold with 7H9 medium to give a BCG suspension with OD_600_ of ∼0.3, with 2% DMSO, 0.05% tyloxapol and the desired final concentration of glycolipid (i.e., ranging from 0 up to 20 µg/ml, as indicated). The cultures were then grown for 48 hours at 37°C with shaking to mid-log phase (OD_600_ = ∼0.6). Bacteria were harvested and washed twice with 10 ml of PBS+0.05% tyloxapol, followed by resuspension in PBS+0.05% tyloxapol to the desired concentration. Mouse immunizations were done using the indicated number of CFU of glycolipid modified bacteria in a volume of 0.1 ml, and CFU numbers were calculated based on OD_600_ values with a value of 1 equal to 3×10^8 ^CFU/ml.

### Mammalian cell culture and in vitro infections

Murine cells were cultured in at 37°C in a 5% CO_2_ incubator in RPMI-1640 medium supplemented with 0.8 mM L-glutamine, 10 mM HEPES, 50 µM 2-mercaptoethanol (all from Life Technologies), and 10% fetal calf serum (Gemini). Murine bone marrow-derived dendritic cells (BMDCs) from 6–8 weeks old female C57BL/6 mice (Jackson Laboratories) were generated and cultured as described previously [51]. The murine iNKT hybridomas DN3A4-1.2 and DN32.D3 (both expressing Vα14 and Vβ8.2 TCR chains) were provided by M. Kronenberg (La Jolla Institute for Allergy and Immunology, San Diego, CA) and A. Bendelac (University of Chicago, IL), respectively. BMDCs at a density of 1×10^5^ cells per well in 96 well flat bottom tissue culture plates were infected with BCG strains with or without glycolipid incorporation at the indicated multiplicity of infection (MOI) as previously described [28]. Briefly, BMDCs overlayed with bacteria were centrifuged at 400×g for 10 minutes at 4°C to initiate infection. The infection was continued for 2 hours at 37°C and extracellular bacteria were removed by washing the BMDCs twice with warm complete RPMI media. The infected BMDCs were co-cultured with 5×10^4^ cells per well of iNKT hybridoma for 24 hours at 37°C. Supernatants were collected and assayed for IL-2 by sandwich ELISA.

### Mice and immunization

Six to eight weeks old female C57BL/6 mice (Jackson Laboratories) were purchased and allowed to adapt to local conditions for a minimum of 1 week prior to use in experiments. Human CD1d knock-in mice have been recently described [34], and were maintained in a breeding colony in our facility and used at 6–8 weeks of age. All mice were housed and maintained in specific pathogen-free conditions in compliance with procedures and regulations approved by the Institute for Animal Studies and Biosafety Committee of the Albert Einstein College of Medicine. This study was specifically approved by the Albert Einstein College of Medicine Institutional Animal Care and Use Committee (IACUC). Mice were primed with either unmodified or glycolipid modified rBCG-SIV *gag* i.v. via tail vein or s.c. in the flank region with the indicated bacterial doses diluted in PBS with 0.05% tyloxapol to a final volume of 100 µl. Boosting was done 8 weeks after priming with 10^7^ VP of rAd5-SIV *gag* in 100 µl of PBS injected i.m. route in the quadriceps muscles (50 µl per leg). At the indicated days post boosting, mice were anesthetized to obtain samples of peripheral blood (for time-course measurements), or were sacrificed for removal of spleens.

### Analysis of NKT cell anergy

To determine if glycolipid adjuvant induced NKT cell anergy when administered with recombinant BCG, C57BL/6 mice were injected s.c. with 5×10^6 ^CFU of the glycolipid modified rBCG-SIV *gag* or intraperitoneally (i.p.) with 4 µg of either α-GC or α-C-GC. Seven days later, splenocytes obtained from these mice were analyzed *ex vivo* for recall responses of NKT cells by stimulating 5×10^5^ cells with 100 nM α-GC in 96-well plates for 48 hours, and supernatants were assayed for IFN-γ and IL-4 by sandwich ELISA. Splenic NKT cells, identified by staining with α-GC loaded mCD1d tetramers and anti-CD3 antibody, were also analyzed for PD-1 expression by flow cytometry.

### Assessment of in vivo T cell proliferation by CFSE dilution

Donor splenocytes were obtained from tandem dimer tomato (tdT) red fluorescent protein-transgenic mice expressing ovalbumin-specific OT-I TCR-transgenes (tdT-Tg/OT-I TCR-Tg mice), and CD8^+^ T cells were isolated using a CD8 negative selection kit (Miltenyi Biotech, San Diego, CA). After isolation, CD8^+^ T cells were labeled with 10 µM CFSE (Molecular Probes, Eugene, OR). Recipient human CD1d knock-in mice received 5×10^6^ labeled cells via the lateral tail vein, and were then vaccinated s.c. with 5×10^6 ^CFU of glycolipid-modified or unmodified rBCG-OVA as indicated, or received PBS plus 0.05% tyloxapol as a negative control. Splenocytes were harvested 6 days later, stained with anti-B220-APC and anti-CD8-PerCP, and analyzed using a LSR-II-yellow flow cytometer (BD Biosciences). CFSE fluorescence of the transferred population was determined by gating on the B220^neg^CD8^+^ lymphocytes expressing tdTomato.

### Statistical analysis

All statistical analyses were carried out using non-parametric tests. A Mann-Whitney test was used for pair-wise comparisons, and a Kruskal-Wallis test for multiple comparisons. For post-hoc analysis after Kruskal-Wallis test, we performed a Mann-Whitney U test using the Bonferroni correction to adjust the probability. Values of *P*<0.05 were considered statistically significant. All statistical tests were done using Prism software (GraphPad).

## Supporting Information

Figure S1 **Chemical structure of glycolipids used in this study.** The structures of α-C-GC and 7DW8-5 are similar to that of the parental *O*-glycoside, α-GC. However, in α-C-GC there is an α-anomeric carbon-based glycosidic linkage, whereas in 7DW8-5 there is a shorter fatty amide chain terminating in a *p*-fluorinated benzene ring.
(TIF)

Figure S2 **Representative flow cytometer data to illustrate the gating strategies for identifying the AL11 tetramer positive CD8^+^ T cell population.** Forward (FSC-A) and side (SSC-A) scatters were used to gate T lymphocyte population. Dead cells were excluded from Blue Live/Dead staining followed by exclusion of non-singlet events using forward side scatters (FSC-A and FSC-H). CD8^+^ T cells were gated from the CD8^+^ B220^neg^ population and analyzed for AL11 tetramer staining.
(TIF)

Figure S3 **Kinetics of serum cytokine responses to free glycolipids.** IFN-γ and IL-4 responses in mice during 48 hour time period following the intraperitoneal injection of 4 nmoles of free glycolipids. Control mice received saline injections. Median values with interquartile ranges are shown for groups of three mice sampled at each time point.
(TIF)
