# Supplementary figures and images for: Improving Mycobacterium bovis Bacillus Calmette-Guèrin as a Vaccine Delivery Vector for Viral Antigens by Incorporation of Glycolipid Activators of NKT Cells
Source: PLoS One. 2014 Sep 25;9(9):e108383. doi: 10.1371/journal.pone.0108383 (PMC4177913; doi:10.1371/journal.pone.0108383)

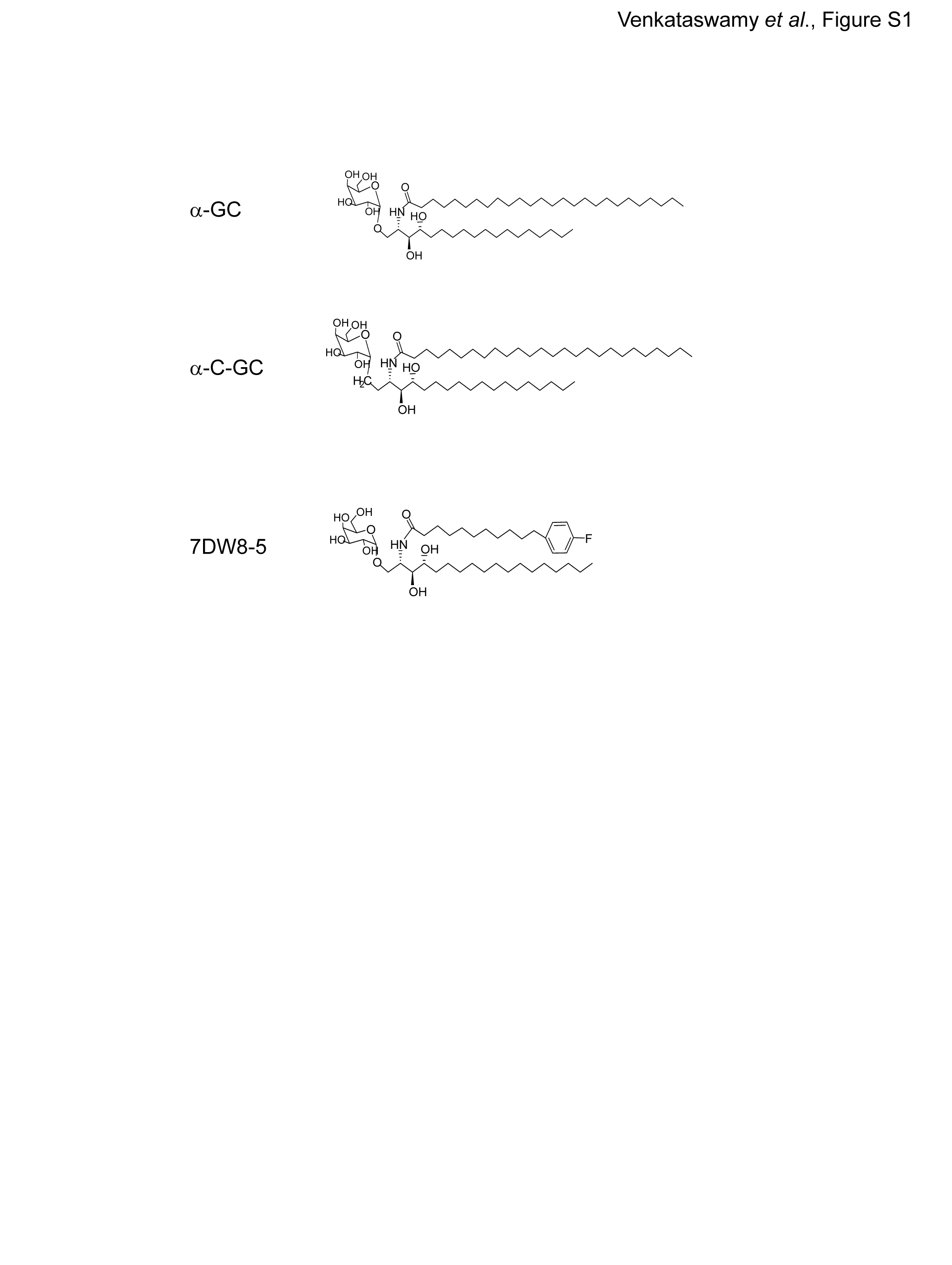

Supplement: Figure S1 — Chemical structure of glycolipids used in this study. The structures of α-C-GC and 7DW8-5 are similar to that of the parental O-glycoside, α-GC. However, in α-C-GC there is an α-anomeric carbon-based glycosidic linkage, whereas in 7DW8-5 there is a shorter fatty amide chain terminating in a p-fluorinated benzene ring. (TIF) [file pone.0108383.s001.tif]

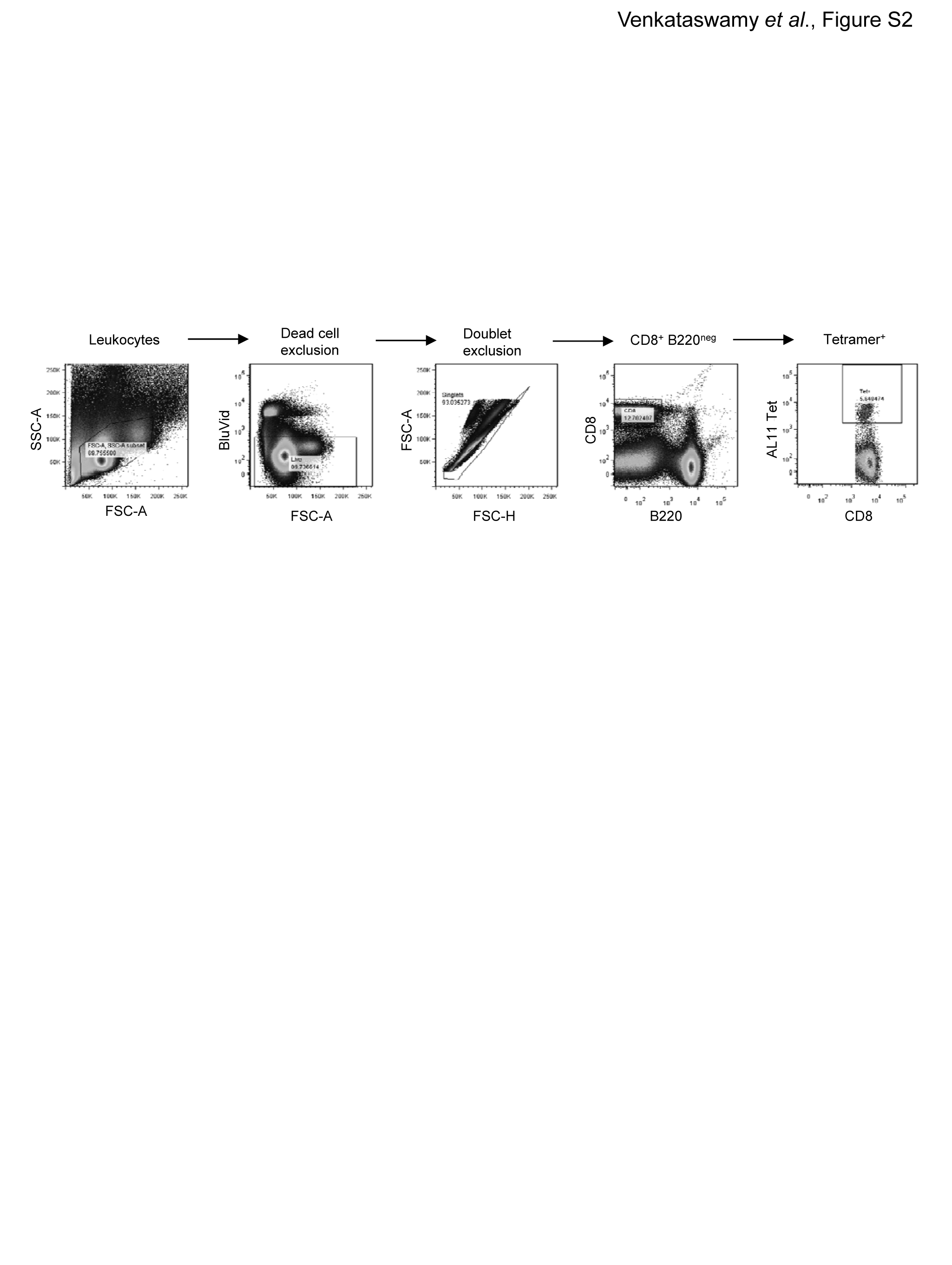

Supplement: Figure S2 — Representative flow cytometer data to illustrate the gating strategies for identifying the AL11 tetramer positive CD8+ T cell population. Forward (FSC-A) and side (SSC-A) scatters were used to gate T lymphocyte population. Dead cells were excluded from Blue Live/Dead staining followed by exclusion of non-singlet events using forward side scatters (FSC-A and FSC-H). CD8+ T cells were gated from the CD8+ B220neg population and analyzed for AL11 tetramer staining. (TIF) [file pone.0108383.s002.tif]

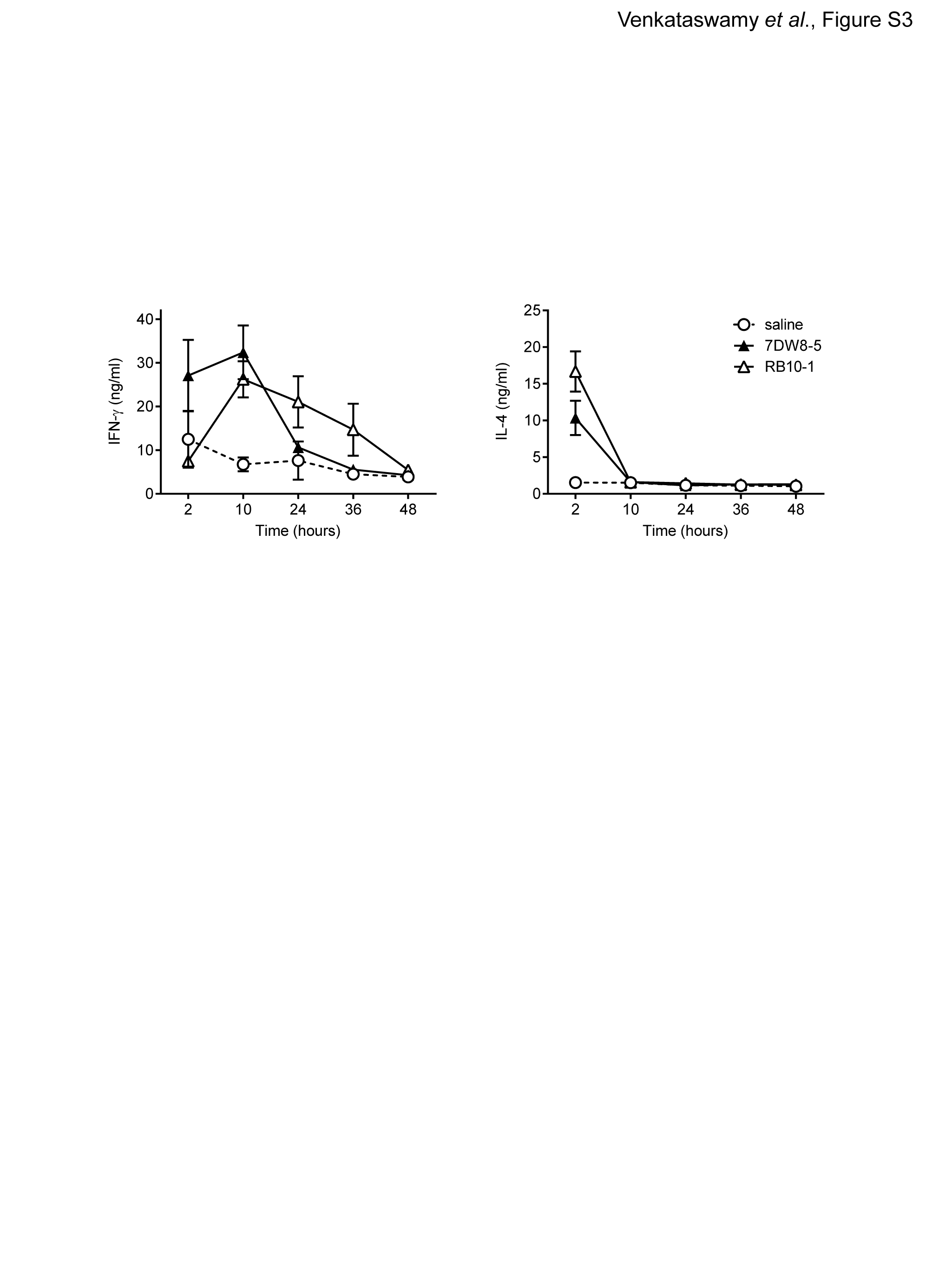

Supplement: Figure S3 — Kinetics of serum cytokine responses to free glycolipids. IFN-γ and IL-4 responses in mice during 48 hour time period following the intraperitoneal injection of 4 nmoles of free glycolipids. Control mice received saline injections. Median values with interquartile ranges are shown for groups of three mice sampled at each time point. (TIF) [file pone.0108383.s003.tif]
